# THE ASSOCIATION BETWEEN COGNITIVE FUNCTION AND TAI-CHI: FINDINGS FROM THE PINE STUDY

**DOI:** 10.1093/geroni/igz038.3004

**Published:** 2019-11-08

**Authors:** Ying-Yu Chao, Ying-Yu Chao, Peijia Zha, Xinqi Dong

**Affiliations:** 1 Rutgers School of Nursing, Newark, United States; 2 Rutgers School of Nursing, Newark, New Jersey, United States; 3 Rutgers Institute for Health, Health Care Policy and Aging Research, New Brunswick, New Jersey, United States

## Abstract

The study examined the association between cognitive function and Tai Chi practice among older Chinese Americans. Data from the Population Study of Chinese Elderly in Chicago (N=3,157) was analyzed by multivariate logistic regression. Cognitive function was assessed by global cognition, episodic memory, executive function, working memory, and Chinese Mini-Mental State Examination (C-MMSE). The results showed that cognitive function was significantly associated with Tai Chi practice. Participants with higher scores of global cognition (OR = 1.44, 95% CI: 1.20–1.73, p = 0.00), episodic memory (OR =1.27, 95% CI: 1.10–1.47, p = 0.00), executive function (OR = 1.017, 95% CI:1.00–1.03, p = 0.01), working memory (OR = 1.07, 95% CI: 1.02–1.12, p = 0.01), and C-MMSE (OR = 1.06, 95% CI: 1.03–1.09, p = 0.00) were more likely to practice Tai Chi. This study demonstrated that Tai Chi may benefit cognitive function in Chinese older adults.

